# Semi-empirical model for Henry’s law constant of noble gases in molten salts

**DOI:** 10.1038/s41598-024-60006-9

**Published:** 2024-06-04

**Authors:** Kyoung O. Lee, Wesley C. Williams, Joanna McFarlane, David Kropaczek, Dane de Wet

**Affiliations:** Oak Ridge National Laboratory, 1 Bethel Valley Road, Oak Ridge, TN 37831-6368 USA

**Keywords:** Thermodynamics, Materials science, Chemistry, Chemical engineering, Physical chemistry

## Abstract

Henry’s law constant, which describes the proportionality of dissolved gas to partial pressure of free gas in liquid–gas equilibrium systems, can also be applied to mass transport applications. In this work, we investigated an approach for determining the solubility of noble gases in a molten salt liquid utilizing the equilibrium concept of Henry’s gas constant. *Henry’s gas constant* is described as a mathematical function dependent on the van der Waals radius of the noble gas and the temperature of the molten salt. The alteration in Gibbs free energy encompasses contributions from both surface and volume energies. Enthalpy and entropy are deduced from these surface and volume energies in the Gibbs free energy formulation. A comparative analysis was conducted between the conventional method and our proposed model. Moreover, useful chemical properties can be determined from examination of surface and volume energies. Our findings provide an accurate and general theory of Gibbs free energy that can be validated experimentally based on the model proposed herein. This work unifies the prediction of Henry gas constant and subsequently the entropy and enthalpy calculation for noble gases in a molten salt solution to a single functional form using van der Waals radius of the gas and temperature of the system. This functional form is then used to perform a multiple regression method to find two parameters corresponding to the surface energy and volume energy. These two parameters are consistent between all combinations of noble gas and molten salt.

## Introduction

This study investigated the partitioning of noble gases between solution in the molten salt liquid phase and the cover gas. Noble gases constitute the largest fraction of volatile fission products, so their ability to move into the gas phase affects the operational performance of molten salt reactors (MSRs)^1,2^. Therefore, mass transport of radioactive species produced during nuclear fission is essential to understanding the performance and the control of molten fuel salts in MSRs. Particularly in liquid–gas systems, the mass transfer of noble gases from the liquid to the gaseous phase across the entire loop system must be taken into account to determine the reactivity and other key nuclear reactor performance parameters.

The system under consideration can be defined as a liquid phase in which a noble gas with limited solubility dissolves in a molten salt. The behavior of radioactive species in molten salts within molar salt reactors can be described by a combination of thermochemical equilibrium and transport dynamics that depend on thermophysical properties in well-mixed turbulent flows. For molten salts, under sufficient conditions of temperature above 700 °C, noble gas solubility is temperature dependent. Additionally, the solubility of a gas dissolved liquid generally is associated with both static and dynamic fluid states. Experimentally, the solubility of gas in a liquid is measured under conditions without any flow. For noble gases in particular, it can be assumed that the liquid–gas transition is governed by species stability expressed as Gibbs free energy.

Henry’s law constant describes the equilibrium ratio of liquid-to-vapor concentrations^3^. Therefore, a study of the temperature dependence of Henry’s law constant was performed in which Gibbs free energy data were fit by applying a regression equation using the salt’s surface tension and the atomic size of noble gases. Quantitative analysis by multiple successive regressions achieved results that strongly support this approach. The results compared favorably with Henry’s law constant data collected from historical references for the molten salt mixtures with noble gases. The computed Henry’s law values depend on salt-specific data, such as surface tension, that have been measured only for a few specific molten salt mixtures.

**Figure 1:**
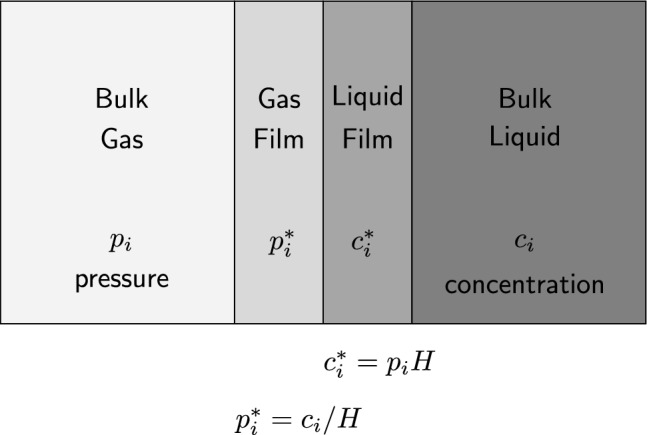
The understanding of the gas–liquid interface is clarified by the two-film theory, which elucidates this phenomenon by utilizing partial pressures and concentrations, where *H* represents Henry’s law constant.

The two-film theory has been used to analyze liquid–gas mass transport. Figure 1 is a diagram demonstrating the assumed layered interfaces between the gas and liquid phases. The mass transfer process is considered to occur at the interface between the gas film side and the liquid film side. In the figure, $c_i$ represents the concentration of species in the liquid phase (i.e., dissolved species), whereas $p_i^*$ corresponds to the partial pressure of species in the gas phase (i.e., bubble behaviors). During the phase transition, a gas film and a liquid film are formed on the surface between liquid and gas. When the gas transitions from liquid to gas, the behavior is dependent on the concentrations at the interface, $c_i^*$ in the liquid film and $p_i^*$ in the gas film. The two-film theory is a diffusion-based mechanism that can be used to describe the transport of gaseous masses across a liquid film using Henry’s gas constant. Henry’s gas constant plays a pivotal role in this context, and it is essential to explore the complexity of these mechanisms and to establish a comprehensive understanding of these phenomena.

## Methods

When the temperature of the system changes, Henry’s constant changes for the equilibrium process and can be described by the Gibbs free energy. In general, Gibbs free energy is a chemical potential that can be used to determine the stable chemical species of systems and their phase behavior. For noble gas behavior, the equilibrium of a system is established through transitions between liquid and gas phases until the lowest energy condition is met. Thus, models based on Gibbs free energy offer essential insight into the conditions necessary for liquid–gas mixing during phase transition processes, describing surface effects and volumetric energies at specific temperatures and pressures. The temperature of the thermodynamic system remains constant during the transition. However, because the solute and solvent have different densities, the total volume of the system changes with the relative amounts of solute and solvent—that is, during a phase change. The entropy change for an equilibrium process can be explained by the Gibbs free energy change (in J/mol), $\Delta G=\Delta H -T\Delta S$, where $\Delta H$ (in J/mol) represents the enthalpy change, $\Delta S$ (in J/mol  K) denotes the entropy change, and *T* (in K) stands for the temperature in Kelvin within the system. In this study, we assume that the noble gas phase transition process consists of a temperature-dependent process and a van der Waals radius that has a polarization effect^4,5^. For the solute, a noble gas, to spontaneously separate from the solvent, which takes the form of a molten salt, the translational energy of a solute atom must surpass the intermolecular binding forces trapping it in the salt. The enthalpy change of this process is denoted as $\Delta H$, and the reaction consistently exhibits an endothermic nature ($\Delta H$ > 0), owing to the energy necessary to overcome the interactions among the molecules within the molten salt.

The corrected Henry’s gas constant for a gas within a liquid can be explained through the equilibrium between the Gibbs free energies of two concurrently existing phases. The phase transition is represented by $A(g) \rightleftharpoons A(l)$, where *A* is the concentration of a noble gas and *l* denotes the gas dissolved in the solution while *g* indicates the bubbles in the solution. The equilibrium exists between the gas phase and the liquid (dissolved) phase, including during the reverse process. When examining the scenario where a mole of noble gas dissolves in a molten salt fluid at constant temperature, *T*, the Gibbs free energy equation results in the following:

1 $$\begin{aligned} K_H(r,T;\gamma (T),\alpha , \beta , K_{H}^0)&=\exp {\left( \frac{ \Delta G}{RT} \right) } \end{aligned}$$

Note that the corrected Henry’s gas constant, $K_H$, is defined as the dimensionless ratio of $K_H^e$ [mole/(cm^2^ atm)] divided by $K_{H}^0$ [mole/(cm^2^ atm)], where $K_H^e$ corresponds to the experimental data and $K_{H}^0$ represents the reference value with $\alpha$ and $\beta$ as empirical constants . In experimental data where the impact of pressure can be effectively averaged, alterations in the temperature of a system lead to modifications in Henry’s constant. This change is directly connected to variations in Gibbs free energy. Employing the method of least squares regression facilitates the determination of $\alpha$ and $\beta$, which are used to compute the equilibrium constant. The units of $\alpha$ and $\beta$ can be adjusted accordingly to convert the Gibbs energy change to joules per mole. Equation (2) establishes a connection between Henry’s law constant and the product of the liquid’s surface tension and the van der Waals radius, along with the volume energy, all in relation to temperature.

For a semi-empirical model, a relationship between Henry’s law constants and the energy properties that characterize the noble gas transition between gas and dissolved gas states is established. This encompasses the capability of the model to analyze the contribution to the Gibbs energy of two components: the volumetric free energy and the surface free energy of both gas molecules and the solvent. The overall change in the Gibbs free energy ($\Delta G =\Delta G_{\gamma } + \Delta G_v$, ) of a component system can include contributions from both surface and volume energies, as given by the following expression:

2 $$\begin{aligned} \Delta G(r,T;\gamma (T),\alpha , \beta )&= RT \ln {\left( K_H\right) }=4\pi r^2 \alpha \gamma (T) + \displaystyle \frac{4}{3}\pi r^3 \beta RT, \end{aligned}$$

where $R=$ 8.3145  [J/(mol K)] is the ideal gas constant, *r* is the van der Waals radius in angstrom units, and *T* is temperature in $\text {K}$. The surface tension (in erg/cm^2^) of a liquid salt decreases linearly with the change in temperature while maintaining a constant pressure, given by the expression

3 $$\begin{aligned} \gamma (T) =\frac{\partial \gamma (T)}{\partial T} \left( T-273.15\right) +\gamma _0 . \end{aligned}$$

The enthalpy change is determined to be independent of temperature, as evidenced by

4 $$\begin{aligned} \Delta {H}&= RT^2 \frac{d\ln {\left( K_H\right) }}{dT} = -4\pi \alpha r^2 \gamma _0 \end{aligned}$$

The entropy change is obtained from the relationship between Gibbs free energy and enthalpy as follows:

5 $$\begin{aligned} \Delta S&=-8\pi \alpha r^2 \frac{\gamma (T)}{T} +4\pi \alpha r^2 \frac{\partial \gamma (T)}{\partial T} -\displaystyle \frac{4}{3}\pi r^3 \beta R . \end{aligned}$$

### Rights and permissions

This manuscript has been authored by UT-Battelle LLC under contract DE-AC05-00OR22725 with the US Department of Energy (DOE). The US government retains and the publisher, by accepting the article for publication, acknowledges that the US government retains a nonexclusive, paid-up, irrevocable, worldwide license to publish or reproduce the published form of this manuscript, or allow others to do so, for US government purposes. DOE will provide public access to these results of federally sponsored research in accordance with the DOE Public Access Plan (http://energy.gov/downloads/doe-public-access-plan).

## Results

**Figure 2:**
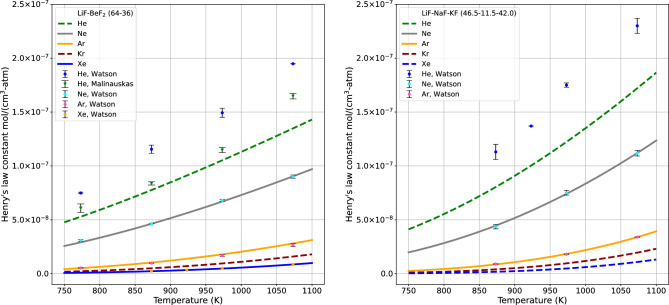
The change in Gibbs free energy in 2LiF–${{\hbox {BeF}}_{2}}$ system has been amended to include the appropriate correction for Henry’s gas constant. This corrected equation is then utilized in a nonlinear regression approach that takes into account both the surface and volume energy terms of the Gibbs energy. Equation (2) considers temperature variations and the van der Waals radii of noble gases. The measured solubilities of neon, argon, and xenon gases play a crucial role as input data points for generating the regressed parameters. By employing nonlinear regression analysis with the entirety of this data, it was possible to predict helium and krypton values’ solubilities in 2LiF–${{\hbox {BeF}}_{2}}$. Additionally, the analysis allowed for the prediction of helium, krypton, and xenon in LiF–NaF–KF. These projections give clearly defined $\alpha$ and $\beta$ parameters that are inherent to 2LiF–${{\hbox {BeF}}_{2}}$ and LiF–NaF–KF salts.

The solubility of noble gases by Henry’s gas constant were calculated as a function of temperature in a molten salt mixture, two eutectic mixtures of 2LiF–${{\hbox {BeF}}_{2}}$ (Flibe) and and LiF–NaF–KF (Flinak). This semi-empirical method to calculate $\alpha$ and $\beta$ parameters assumes that the noble gas phase evaporative process is temperature-dependent. Henry’s law constants for helium, neon, argon, krypton, and xenon in 2LiF–${{\hbox {BeF}}_{2}}$ (64–36 mol%) and LiF–NaF–KF (46.5–11.5–42.0 mol%) solutions at various temperatures were used to benchmark gas solubility in this study. These constants were utilized in the regression fit of the data. Figure 2 represents the change in Henry’s law constant with temperature.

The objective of this analysis was to ascertain $\alpha$, $\beta$, and $K_H^0$ values by the regression in Table 1, and each set of values was shown to remain constant for all five noble gases dissolved in 2LiF–${{\hbox {BeF}}_{2}}$ and LiF–NaF–KF along with the corresponding surface tension. In Flibe, $\beta$ is negative, while in Flinak, $\beta$ is positive. Flinak is composed exclusively of alkali fluorides that behave as separate ions in the melt. In FLibe, the characteristic of a negative $\beta$ arises from the behavior of beryllium fluoride in the melt. The chemistry of beryllium, being an alkaline earth, is different from that of the alkali metals. ${{\hbox {BeF}}_{2}}$ is nonionic and associates in solution, and this property is ultimately responsible for its high viscosity. The melting point of ${{\hbox {BeF}}_{2}}$ is 827 (in K), which is lower than NaF 1266 (in K) and KF 1131 (in K)^6^. As long as the salt temperature remains above the melting point of ${{\hbox {BeF}}_{2}}$, this volume energy contribution is negative ($\Delta G_v<0$). However, when the salt temperature falls below the melting point of NaF and KF, this volume energy contribution becomes positive ($\Delta G_v>0$). Simultaneously, van der Waals radius values were incorporated; the model regression effectively fits the experimental data (with the exclusion of all helium temperature point in the Fig. 2). Discrepancies between the model and all helium data point are likely due to polarization effects at the subatomic level caused by the helium only having duet electrons in the full outer shell as opposed to the octet shells of the larger gases. This means that helium has a less dramatic polarization due to London dispersion forces^7^. Also, in helium, the experimental data did not align in 2LiF–${{\hbox {BeF}}_{2}}$^8,9^. However, a notable level of agreement was apparent from the fit of the Henry’s law data for neon, argon, and xenon in $2\hbox {LiF}-{{\hbox {BeF}}_{2}}$ on the left side of Fig. 2, as well as for neon and argon in LiF–NaF–KF on the right side of Fig. 2. The model predictions were employed to generate a dataset to conduct a comparative analysis between the noble gases experimental measurements. The model was used to predict the Henry’s law constant for both helium and krypton ($2\hbox {LiF}-{{\hbox {BeF}}_{2}}$) and helium, krypton, and xenon (LiF–NaF–KF), as shown in Fig. 2; data were unavailable for the latter.

**Table 1:** The regression model’s parameters, including R-squared and reduced chi-square and surface tension erg/cm^2^ (ref.^8^) are presented.

Parameter	2LiF–${{\hbox {BeF}}_{2}}$	LiF–NaF–KF
$R^2$	0.9988	0.9841
$\chi ^2_\mathrm{\nu }$	144.2	162.4
$\alpha$	− 3.3584 ± 0.0645	− 4.6541 ± 0.0938
$\beta$	− 0.0215 ± 0.0016	0.0105 ± 0.0023
$K_H^0$	7.8622$\times 10^{-7}$ ± 0.2190 $\times 10^{-7}$	1.4246 $\times 10^{-6}$ ± 0.0644 $\times 10^{-6}$
Equation (3)	2LiF–${{\hbox {BeF}}_{2}}$	LiF–NaF–KF
$\displaystyle \partial \gamma (T)/\partial T$	− 0.09	− 0.0788
$\gamma _0$	235.5	237.0

The reduced chi-squared value is obtained by dividing the chi-squared value by the degrees of freedom ($\nu$).

**Figure 3:**
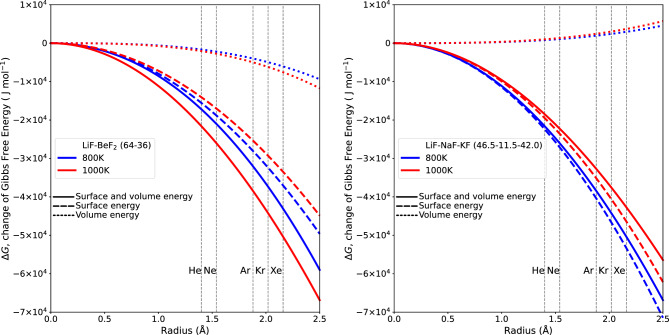
The change in Gibbs free energy within the 2LiF− ${{\hbox {BeF}}_{2}}$ and LiF–NaF–KF system is closely tied to the van der Waals radius. The radius for each noble gas in the system involves an evaluation of the two key components of Gibbs free energy analysis: volume energy and surface energy. Volume energy gives the potential for a phase transition—either evaporation or condensation—to occur. Whereas surface energy quantifies the energy necessary for interface formation. The surface and volume energies are clearly shown along with the cumulative sum.

**Figure 4:**
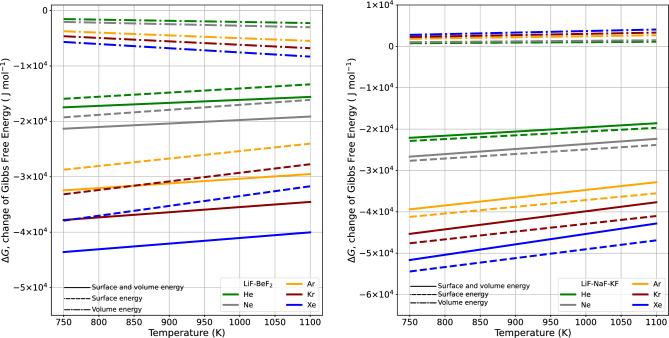
The volume and surface energy functions change directly in relation to the cube of the van der Waals radius and the square of the van der Waals radius, respectively. Both volume energy and surface energy are shown separately, and their combined total is also illustrated in 2LiF–${{\hbox {BeF}}_{2}}$ and LiF–NaF-KF.

**Figure 5:**
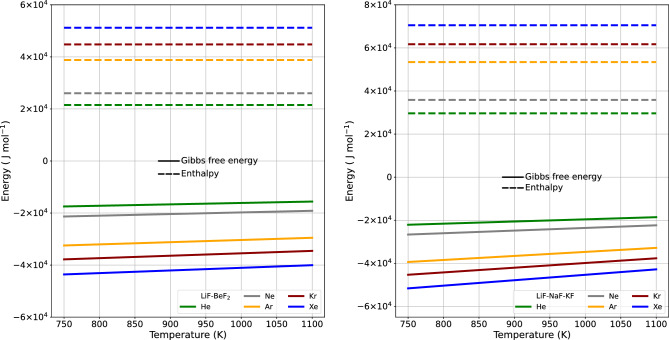
The graph reveals the temperature dependency of $\Delta G$ in the vaporization of noble gases. $\Delta G$ varies with temperature, whereas $\Delta H$ remains constant. As expressed by $\Delta G = \Delta H - T \Delta S$, at this specific temperature, $\Delta G$ is less than 0, $\Delta H$ is greater than 0, and $T \Delta S$ is greater than 0. As a result, noble gases undergo spontaneous evaporation from 2LiF–${{\hbox {BeF}}_{2}}$ and and LiF–NaF–KF. The enthalpy of solution indicates the quantity of heat absorbed. This reaction is consistently endothermic ($\Delta H > 0$) due to the necessity of sufficient energy to escape the interactions at a constant pressure throughout the dissolution process. The increase in temperature effectively can be seen as adding the energy required to increase the gas to liquid contact surface area.

Figure 3 shows how the change in Gibbs free energy, as determined by the interplay of surface energy and volume energy from Eq. (2), depends on the value of the van der Waals radius on the x-axis. The individual contributions of surface energy and volume energy can be observed, and the cumulative amount is displayed. The surface energy has a greater influence than the volume energy. In all cases, the Gibbs energy change ($\Delta G$) for each noble gas exhibited a decrease with increased radius. Consequently, thermodynamic equilibrium within the molten state leads to a feedback release of energy, facilitating rapid vapor formation and driving the system toward the lowest possible Gibbs energy state. Much like the interactions of surface and volume energies, Fig. 4 illustrates the change of $\Delta G$ with temperature. As the temperature increases, in Eq. (2), the surface energy increases and the volume energy decreases, but the total contribution increases.

**Figure 6:**
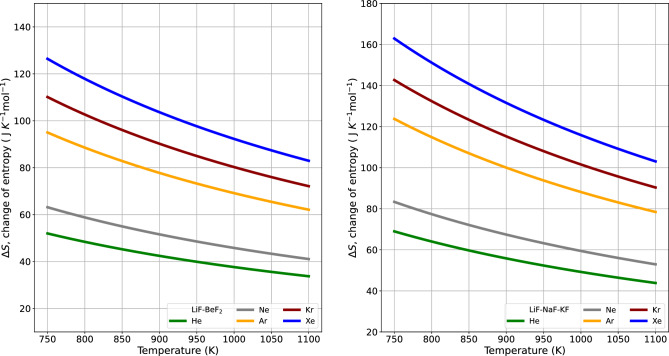
The spontaneity of a reaction depends on changes in both enthalpy and entropy. This is highlighted by the invariant nature of $\Delta H$ coupled with decreasing $\Delta S$ with increasing temperature. This pattern corresponds to increasing values of Henry’s gas constant in 2LiF–${{\hbox {BeF}}_{2}}$ and LiF–NaF–KF.

**Table 2:** Enthalpy contributions to the Gibbs free energy of both surface and volume energies were compared using the conventional method.

		2LiF-${{\hbox {BeF}}_{2}}$				LiF–NaF–KF			
	van der Waals	Model	$\ln (K_H^e)$			Model	$\ln (K_H^e)$		
Noble gas	radius (Å) (ref.^10^)	$\Delta H$	$\Delta H$	$\Delta S$	RPD	$\Delta H$	$\Delta H$	$\Delta S$	RPD
He	1.40	21513.2	21657.2^8^	108.3	0.7	29635.0	28153.6^8^	100.7	5.1
			22568.9^9^	109.3	4.8	–	–	–	–
Ne	1.54	26031.0	24802.4^8^	111.8	4.8	35858.4	36746.6^8^	98.8	2.4
Ar	1.88	38794.0	36707.8^8^	111.0	5.5	53439.8	51669.7^8^	95.0	3.4
Kr	2.02	44787.0	–	–	–	61695.3	–	–	–
Xe	2.16	51210.2	51630.4^8^	106.1	0.8	70543.4	–	–	–

The model from Eq.  (1) using surface tension from Eq.  (3). The regression model for $\ln (K_H^e)$ is characterized by two conditions: one without the presence of krypton (2LiF–${{\hbox {BeF}}_{2}}$) and another with neither krypton nor xenon (LiF–NaF–KF) the van der Waals radius of the noble gas and R, the gas constant 8.314 $\text{J}\cdot \text{K}^{-1}\cdot \text{mol}^{-1}$ . $\Delta H$ is expressed in units of $\text{J}\cdot \text{mol}^{-1}$ and $\Delta S$ is measured in units of $\text{J}\, \text{K}^{-1}\cdot \text{mol}^{-1}$.

From the Gibbs equation, it can be seen if the change in Gibbs energy is negative and the change in enthalpy and entropy will be positive, the transition is an endothermic process. Figure 5 shows the change in Gibbs free energy and enthalpy as a function of temperature for noble gases. In most reactions, enthalpy is associated with the heat capacity as outlined in Eq. (4), and it remains relatively stable across varying temperatures. Entropy from Eq. (5) is the difference between Gibbs energy and enthalpy change, as shown in Fig. 6. The literature showed enthalpy change and entropy change from the $K_H^e$ with temperature^8^. Conventionally, the natural logarithm of $K_H^e$ is plotted against the inverse of temperature (1/*T*) and therefore a linear regression can be used to determine changes in enthalpy and entropy. The resulting straight line has a slope of $-\Delta H/R$ and an intercept of $\Delta S/R$, as shown in Table 2. Table 2 presents a comparison of values. The enthalpy value is compared to both the reference method or experimental data and our model. The relative percent difference (RPD), the absolute difference value divided by the mean, is within 5.5%. Based on the van der Waals radius, the noble gases’ elements exhibit distinct effects on the parameters $\alpha$, $\beta$ and $K_H^0$. These parameters are intricately tied to the interaction of the dissolved gas molecules’ radius with the ionic environment within the melt. An exponential relationship exists between Henry’s law constant and the Gibbs energy $\Delta G$. Although $\Delta G$ is associated with surface energy^8,11^, this model was introduced to incorporate Gibbs volumetric energy. The enthalpy term of the $\ln {K_H^e}$ method and our model remains constant at a given temperature. The entropy of the model varies with temperature while the $\ln {K_H^e}$ method remains constant, making comparison difficult.

## Conclusions

This paper establishes connections among several physicochemical parameters by effectively linking them to contributions to the Gibbs energy of gas transport through a two-layer film interface. Surface and volume terms for Gibbs free energies were used to show enthalpic and entropic trends. The findings demonstrate significant alignment with pertinent experimental data. As a result, forecasts for Henry’s gas constant values pertaining to helium, krypton, or xenon were formulated, establishing a crucial foundation for future experimental applications and theoretical inquiries. The Gibbs free energy associated with liquid–gas interfaces and volumes is determined by the van der Waals radius of each noble gas. In this work, we introduce a refined and comprehensive extension of this theory. Notably, our findings reveal that as temperature rises, noble gases situated in the interfacial region between liquid and gas phases exhibit a noteworthy volumetric energy contribution.

### Supplementary Information

Supplementary Information.

## Data Availability

All data generated or analyzed during this study are included in this published article and its Supplementary Files.

## References

[1] McFarlane J (2023). The effect of interfacial phenomena on gas solubility measurements in molten salts. Front. Energy Res..

[2] Field PE (1975). Gas solubility in molten salts. Adv. Molten Salt Chem..

[3] Sander R (2014). Compilation of Henry’s law constants, version 3.99. Atmos. Chem. Phys. Discuss.

[4] Mantina M, Chamberlin AC, Valero R, Cramer CJ, Truhlar DG (2009). Consistent van der Waals radii for the whole main group. J. Phys. Chem. A.

[5] Simonin J-P (2011). Effect of polarization on the solubility of gases in molten salts. J. Chem. Phys..

[6] Chase, M. W. Jr. Janaf thermochemical tables. *J. Phys. Chem. Ref Data* (1985).

[7] Fedorov DV, Sadhukhan M, Stöhr M, Tkatchenko A (2018). Quantum-mechanical relation between atomic dipole polarizability and the van der Waals radius. Phys. Rev. Lett..

[8] Watson G, Evans R, Grimes W, Smith N (1962). Solubility of noble gases in molten fluorides. in lif-bef2. J. Chem. Eng. Data.

[9] Malinauskas AP, Richardson DM (1974). The solubilities of hydrogen, deuterium, and helium in molten li2bef4. Ind. Eng. Chem. Fundam..

[10] Bondi A (1964). van der Waals volumes and radii. J. Phys. Chem..

[11] Lee A, Johnson E (1969). Prediction of gas solubility in molten salts. Ind. Eng. Chem. Fundam..

